# Entropy-Based GLDS Method for Social Capital Selection of a PPP Project with q-Rung Orthopair Fuzzy Information

**DOI:** 10.3390/e22040414

**Published:** 2020-04-07

**Authors:** Li Liu, Jiang Wu, Guiwu Wei, Cun Wei, Jie Wang, Yu Wei

**Affiliations:** 1 School of Economics, Sichuan University, Chengdu 610065, China; llai2mm@163.com; 2 School of Statistics, Southwestern University of Finance and Economics, Chengdu 611130, China; wujiang@swufe.edu.cn (J.W.); weicun1990@163.com (C.W.); 3 School of Business, Sichuan Normal University, Chengdu 610101, China; weiguiwu1973@sicnu.edu.cn (G.W.); JW970326@163.com (J.W.); 4 School of Finance, Yunnan University of Finance and Economics, Kunming 650221, China

**Keywords:** multiple attribute group decision-making (MAGDM), GLDS model, entropy, social capital selection, public–private-partnership (PPP) projects

## Abstract

The social capital selection of a public–private-partnership (PPP) project could be regarded as a classical multiple attribute group decision-making (MAGDM) issue. In this paper, based on the traditional gained and lost dominance score (GLDS) method, the q-rung orthopair fuzzy entropy-based GLDS method was used to solve MAGDM problems. First, some basic theories related to the q-rung orthopair fuzzy sets (q-ROFSs) are briefly reviewed. Then, to fuse the q-rung orthopair fuzzy information effectively, the q-rung orthopair fuzzy Hamacher weighting average (q-ROFHWA) operator and q-rung orthopair fuzzy Hamacher weighting geometric (q-ROFHWG) operator based on the Hamacher operation laws are proposed. Moreover, to determine the attribute weights, the q-rung orthopair fuzzy entropy (q-ROFE) is proposed and some significant merits of it are discussed. Next, based on the q-ROFHWA operator, q-ROFE, and the traditional GLDS method, a MAGDM model with q-rung orthopair fuzzy information is built. In the end, a numerical example for social capital selection of PPP projects is provided to testify the proposed method and deliver a comparative analysis.

## 1. Introduction

In actual decision-making applications, how to choose the most desirable alternative from a given alternative set is very important [1,2,3]. The most useful method involves fusing the assessing information expressed by experts and ranking all alternatives according to the fused results to select the best alternative(s) [4,5,6]. Thus, how to obtain reasonable evaluation information is a valuable topic. To do this, motivated by intuitionistic fuzzy sets (IFSs) [7], Pythagorean fuzzy sets (PFSs) [8] appear to depict the complexity of the evaluation objects. Zhang and Xu [9] defined the Pythagorean fuzzy technique for order preference by similarity to an ideal solution (TOPSIS) to manage multiple attribute decision-making (MADM) issues. Peng and Yang [10] primarily proposed the division and subtraction operations for PFSs. Reformat and Yager [11] solved a collaborative recommender system with PFSs. By connecting the Maclaurin symmetric mean (MSM) [12] operators with PFSs, Yang and Pang [13] defined some novel Pythagorean fuzzy interaction MSM operators. Gou et al. [14] found some important properties of continuous PFSs. Yang et al. [15] designed partitioned Bonferroni mean (PBM) operators for PFSs. Liang et al. [16] studied PFSs based on geometric averaging and BM operators. Ren et al. [17] designed the PF-TODIM model. Liang et al. [18] investigated some BM operators for PFSs. Peng et al. [19] defined some new information measures for MADM problems for PFSs. Furthermore, Yager [20] initially designed the q-rung orthopair fuzzy sets (q-ROFSs), which consisted of the membership degree μ and non-membership degree v, which meets the requirement $\mu^{q}+v^{q}\leq 1.$ Note that q-ROFS can be considered an extension of the IFSs and PFSs, since if q=1, the q-ROFSs reduce to IFSs, and if q=2, the q-ROFSs reduce to PFSs. Liu and Wang [21] developed two aggregation operators to fuse q-ROFSs. Wei et al. [22] defined some new MSM operators for q-ROFSs. Bai et al. [23] built some partitioned MSM operators for q-ROFSs. Liu et al. [24] developed the power MSM operators for q-ROFSs. Liu et al. [25] defined some extended BM operators for q-ROFSs. Liu and Liu [26] designed some BM operators to fuse q-ROFSs. Liu and Liu [27] provided the concept of linguistic q-ROFSs and introduced some power BM operators. Yang and Pang [28] defined partitioned BM operators for q-ROFSs. Wei et al. [29] defined Heronian mean operators for q-ROFSs. Liu et al. [30] also provided some Heronian mean operators to aggregate the q-ROFSs. Wang et al. [31] defined the multi-attributive border approximation area comparison (MABAC) method for multiple attribute group decision-making (MAGDM) using q-ROFSs. 

However, the above-mentioned methods can only rank all alternatives using the score results and failed to reflect the dominance flow of the alternatives over the attributes; on account of this, Wu and Liao [32] proposed the gained and lost dominance score (GLDS) method to solve MADM problems. This method is used to select the most desirable alternative(s) via calculating the dominance flow between any two alternatives with respect to the attributes. The higher the gained dominance score is and the lower the lost dominance score is, the best the alternative will be. Fu et al. [33] also studied the GLDS method under a hesitant fuzzy linguistic setting. Liao et al. [34] proposed the life satisfaction evaluation model in an earthquake-hit area using the PL-GLDS integrated method. According to the above three works, we can see that the GLDS method is robust and effective for solving actual MADM problems. Therefore, motivated by them, we extended the GLDS method to q-ROFSs and built a novel q-rung orthopair fuzzy GLDS decision-making model in this study. In addition, the attribute weights are often partly known or unknown; therefore, to manage this problem, the q-rung orthopair fuzzy entropy (q-ROFE) was developed to determine the attribute weights. Thus, the main novelty and contributions of this study are:

(1) The q-ROFS can extend the application scope of the assessment information, and the q-rung orthopair fuzzy Hamacher weighting average (q-ROFHWA) and q-rung orthopair fuzzy Hamacher weighting geometric (q-ROFHWG) operators, which can consider the interrelationship between q-ROFSs, were proposed based on the Hamacher operations.
(2) The previous works assumed that the attribute weights were known, but this is impossible in a complicated decision-making environment. This study defined the q-ROFE, which considers the similarity part and hesitancy part, and as such, is a useful tool for determining the attribute weights.
(3) The previous works ranked all alternatives by the score results but failed to reflect the dominance flow of the alternatives over the attributes; in this study, we proposed the q-rung orthopair fuzzy entropy-based GLDS method for MAGDM issues, which can overcome this limitation.

The remainder of our article is structured as follows. Section 2 briefly reviews some fundamental theories of q-ROFSs. Section 3 presents the q-rung orthopair fuzzy Hamacher weighting average (q-ROFHWA) operator and the q-rung orthopair fuzzy Hamacher weighting geometric (q-ROFHWG) operator. Section 4 proposes the q-ROFE to determine the attribute weights and discusses some properties of the q-ROFE. Based on the traditional GLDS method, Section 5 builds the q-rung orthopair fuzzy entropy-based GLDS method for MAGDM. Section 6 introduces the MAGDM steps based on the q-rung orthopair fuzzy entropy-based GLDS model. Section 7 proposes the social capital selection of public–private-partnership (PPP) projects with q-ROFSs and compares the developed method with the existing methods. Section 8 concludes the paper with some meaningful remarks.

## 2. Preliminaries

In this part, some basic theories related to q-ROFSs [20] are briefly depicted.

**Definition** **1.** *Assume that Ψ is a fixed set. Then, the q-ROFS is given as [20]:*

(1) $$\Psi =\left\{\langle x,\left(\xi_{\Psi }\left(x\right),\zeta_{\Psi }\left(x\right)\right)\rangle |x\in X\right\}\;$$

*where $\xi_{\Psi }:\;X\to \left[0,1\right]$ indicates the membership degree and $\zeta_{\Psi }:\;X\to \left[0,1\right]$ indicates the non-membership degree of elements x∈X to Ψ, and the membership degree and non-membership degree satisfies:*

(2) $${\left(\xi_{\Psi }\left(x\right)\right)}^{q}+{\left(\zeta_{\Psi }\left(x\right)\right)}^{q}\leq 1,q\geq 1\;$$

*On account of the $\xi_{\Psi }\left(x\right)$ and the $\zeta_{\Psi }\left(x\right)$, the indeterminacy membership degree can be computed using the following equation:*

(3) $$\pi_{\Psi }\left(x\right)=\sqrt[{q}]{1-{\left(\xi_{\Psi }\left(x\right)\right)}^{q}-{\left(\zeta_{\Psi }\left(x\right)\right)}^{q}}\;$$

*Then, based on the above, we named Ψ=(ξ,ζ) as a q-rung orthopair fuzzy number (q-ROFN).*

**Definition** **2.** *Given a q-ROFN Ψ=(ξ,ζ), the score function can be found using [21]:*

(4) $$SC\left(\Psi \right)=\frac{1}{2}\left(1+\xi^{q}-\zeta^{q}\right),\;SC\left(\Psi \right)\in \left[0,1\right]$$

**Definition** **3.** *Given a q-ROFN Ψ=(ξ,ζ), the accuracy function can be found using [21]:*

(5) $$AC\left(\Psi \right)=\xi^{q}+\zeta^{q},AC\left(\Psi \right)\in \left[0,1\right]\;$$

*According to the computation results of the score function SC and the accuracy function AC, the order relation between any two q-ROFNs $\Psi_{i}=\left(\xi_{i},\zeta_{i}\right)\left(i=1,2\right)$, can be derived using the following operation laws.*

**Definition** **4.** *Given any two q-ROFNs $\Psi_{i}=\left(\xi_{i},\zeta_{i}\right)\left(i=1,2\right)$, we can derive the score results of $\Psi_{1}$ and $\Psi_{2}$ as being $SC\left(\Psi_{1}\right)=\frac{1}{2}\left(1+{\left(\xi_{1}\right)}^{q}-{\left(\zeta_{1}\right)}^{q}\right)$ and $SC\left(\Psi_{2}\right)=\frac{1}{2}\left(1+{\left(\xi_{2}\right)}^{q}-{\left(\zeta_{2}\right)}^{q}\right)$, respectively, and the accuracy results of $\Psi_{1}$ and $\Psi_{2}$ as $AC\left(\Psi_{1}\right)={\left(\xi_{1}\right)}^{q}+{\left(\zeta_{1}\right)}^{q}$ and $AC\left(\Psi_{2}\right)={\left(\xi_{2}\right)}^{q}+{\left(\zeta \right)}^{q}$, respectively. If $SC\left(\Psi_{1}\right)<SC\left(\Psi_{2}\right)$, then $\Psi_{1}<\Psi_{2}$. However, if $SC\left(\Psi_{1}\right)=SC\left(\Psi_{2}\right)$, then either (1) if $AC\left(\Psi_{1}\right)=AC\left(\Psi_{2}\right)$, $\Psi_{1}=\Psi_{2}$; or (2) if $AC\left(\Psi_{1}\right)<AC\left(\Psi_{2}\right)$, $\Psi_{1}<\Psi_{2}$ [21].*

**Definition** **5.** *Given three q-ROFNs $\Psi_{1}=\left(\xi_{1},\zeta_{1}\right)$, $\Psi_{2}=\left(\xi_{2},\zeta_{2}\right)$, and Ψ=(ξ,ζ), some basic operations on them are as follows [21]: $\begin{array}{l} (1)\;\Psi_{1}⊕\Psi_{2}=\left(\sqrt[{q}]{{\left(\xi_{1}\right)}^{q}+{\left(\xi_{2}\right)}^{q}-{\left(\xi_{1}\right)}^{q}{\left(\xi_{2}\right)}^{q}},\zeta_{1}\zeta_{2}\right);\\ (2)\;\Psi_{1}⊗\Psi_{2}=\left(\xi_{1}\xi_{2},\sqrt[{q}]{{\left(\nu_{1}\right)}^{q}+{\left(\zeta_{2}\right)}^{q}-{\left(\zeta_{1}\right)}^{q}{\left(\zeta_{2}\right)}^{q}}\right);\\ (3)\;\lambda \Psi =\left(\sqrt[{q}]{1-{\left(1-\xi^{q}\right)}^{\lambda }},\zeta^{\lambda }\right),\lambda >0;\\ (4)\;{\left(\Psi \right)}^{\lambda }=\left(\xi^{\lambda },\sqrt[{q}]{1-{\left(1-\zeta^{q}\right)}^{\lambda }}\right),\lambda >0;\\ (5)\;\Psi^{c}=\left(\zeta ,\xi \right). \end{array}$*

## 3. The q-Rung Orthopair Fuzzy Hamacher Aggregation Operator 

Hamacher operations [35] consist of Hamacher products and Hamacher sums. In the past few years, numerous authors have studied the Hamacher operators [36,37,38]. Zhu and Li [39] developed some novel Hamacher t-norm and t-conorm operators under a hesitant fuzzy linguistic environment. Zhang et al. [40] defined some intuitionistic fuzzy operators based on traditional Heronian mean (HM) operation laws and Hamacher operators. Based on single-valued neutrosophic 2-tuple linguistic variables, Wu et al. [41] defined some Hamacher operators and applied them to MADM issues. Liang et al. [42] defined the Hamacher operators under a linguistic neutrosophic setting and gave an application of evaluating land reclamation schemes. Thus, we find that Hamacher operations are a more useful and meaningful tool for aggregating fuzzy assessment information.

Then, according to the basic operation laws of q-ROFNs and the Hamacher operations, in the following, some new q-rung orthopair fuzzy Hamacher operations are deifned.

**Definition** **6.** *Let γ>0, and given three q-ROFNs $\Psi_{1}=\left(\xi_{1},\zeta_{1}\right)$, $\Psi_{2}=\left(\xi_{2},\zeta_{2}\right)$, and Ψ=(ξ,ζ), then the q-rung orthopair fuzzy Hamacher operation laws could be depicted as:*

(6) $$\Psi_{1}⊕\Psi_{2}=\left(\begin{array}{l} \sqrt[{q}]{\frac{{\left(\xi_{1}\right)}^{q}+{\left(\xi_{2}\right)}^{q}-{\left(\xi_{1}\right)}^{q}{\left(\xi_{2}\right)}^{q}-(1-\gamma ){\left(\xi_{1}\right)}^{q}{\left(\xi_{2}\right)}^{q}}{1-(1-\gamma ){\left(\xi_{1}\right)}^{q}{\left(\xi_{2}\right)}^{q}}},\\ \frac{\zeta_{1}\zeta_{2}}{\sqrt[{q}]{\gamma +(1-\gamma )\left({\left(\zeta_{1}\right)}^{q}+{\left(\zeta_{2}\right)}^{q}-{\left(\zeta_{1}\right)}^{q}{\left(\zeta_{2}\right)}^{q}\right)}} \end{array}\right),\;$$

(7) $$\Psi_{1}⊗\Psi_{2}=\left(\begin{array}{l} \frac{\xi_{1}\xi_{2}}{\sqrt[{q}]{\gamma +(1-\gamma )\left({\left(\xi_{1}\right)}^{q}+{\left(\xi_{2}\right)}^{q}-{\left(\xi_{1}\right)}^{q}{\left(\xi_{2}\right)}^{q}\right)}},\\ \sqrt[{q}]{\frac{{\left(\zeta_{1}\right)}^{q}+{\left(\zeta_{2}\right)}^{q}-{\left(\zeta_{1}\right)}^{q}{\left(\zeta_{2}\right)}^{q}-(1-\gamma ){\left(\zeta_{1}\right)}^{q}{\left(\zeta_{2}\right)}^{q}}{1-(1-\gamma ){\left(\zeta_{1}\right)}^{q}{\left(\zeta_{2}\right)}^{q}}} \end{array}\right),\;$$

(8) $$\begin{array}{l} \lambda \Psi \\ =\left(\sqrt[{q}]{\frac{{\left(1+\left(\gamma -1\right){\left(\xi \right)}^{q}\right)}^{\lambda }-{\left(1-{\left(\xi \right)}^{q}\right)}^{\lambda }}{{\left(1+\left(\gamma -1\right){\left(\xi \right)}^{q}\right)}^{\lambda }+\left(\gamma -1\right){\left(1-{\left(\xi \right)}^{q}\right)}^{\lambda }}},\frac{\sqrt[{q}]{\gamma }{\left(\zeta \right)}^{\lambda }}{\sqrt[{q}]{{\left(1+\left(\gamma -1\right)\left(1-{\left(\zeta \right)}^{q}\right)\right)}^{\lambda }+\left(\gamma -1\right){\left(\zeta \right)}^{q\lambda }}}\right), \end{array}$$

(9) $$\begin{array}{l} \Psi^{\lambda }\\ =\left(\frac{\sqrt[{q}]{\gamma }{\left(\xi \right)}^{\lambda }}{\sqrt[{q}]{{\left(1+\left(\gamma -1\right)\left(1-{\left(\xi \right)}^{q}\right)\right)}^{\lambda }+\left(\gamma -1\right){\left(\xi \right)}^{q\lambda }}},\sqrt[{q}]{\frac{{\left(1+\left(\gamma -1\right){\left(\zeta \right)}^{q}\right)}^{\lambda }-{\left(1-{\left(\zeta \right)}^{q}\right)}^{\lambda }}{{\left(1+\left(\gamma -1\right){\left(\zeta \right)}^{q}\right)}^{\lambda }+\left(\gamma -1\right){\left(1-{\left(\zeta \right)}^{q}\right)}^{\lambda }}}\right). \end{array}$$

**Definition** **7.** *Given a group of q-ROFNs $\Psi_{j}=\left(\xi_{j},\zeta_{j}\right)\left(j=1,2,\ldots ,n\right)$ with a weighting vector $\omega_{j}\left(j=1,2,\ldots ,n\right)$ that meets $0\leq \omega_{j}\leq 1,\sum_{j=1}^{n}\omega_{j}=1$, then the q-rung orthopair fuzzy Hamacher weighted averaging (q-ROFHWA) operator is defined as:*

(10) $$\mathrm{q-ROFHWA}\left(\Psi_{1},\Psi_{2},\ldots ,\Psi_{n}\right)=\omega_{1}\Psi_{1}⊕\omega_{2}\Psi_{2}\ldots ⊕\omega_{n}\Psi_{n}=\overset{n}{\underset{j=1}{⊕}}\omega_{j}\Psi_{j}\;$$

*and the q-rung orthopair fuzzy Hamacher weighted geometric (q-ROFHWG) operator is defined as:*

(11) $$\mathrm{q-ROFHWG}\left(\Psi_{1},\Psi_{2},\ldots ,\Psi_{n}\right)={\left(\Psi_{1}\right)}^{\omega_{1}}⊗{\left(\Psi_{2}\right)}^{\omega_{2}}\ldots ⊗{\left(\Psi_{n}\right)}^{\omega_{n}}=\overset{n}{\underset{j=1}{⊗}}{\left(\Psi_{j}\right)}^{\omega_{j}}\;$$

**Theorem** **1.** *Given a group of q-ROFNs $\Psi_{j}=\left(\xi_{j},\zeta_{j}\right)\left(j=1,2,\ldots ,n\right)$, then the fused results using the q-ROFHWA and q-ROFHWG operators are also a q-ROFN where:*

(12) $$\begin{array}{l} \mathrm{q-ROFHWA}\left(\Psi_{1},\Psi_{2},\ldots ,\Psi_{n}\right)=\overset{n}{\underset{j=1}{⊕}}\omega_{j}\Psi_{j}\\ =\left(\begin{array}{l} \sqrt[{q}]{\frac{\prod_{j=1}^{n}{\left(1+\left(\gamma -1\right){\left(\xi_{j}\right)}^{q}\right)}^{w_{j}}-\prod_{j=1}^{n}{\left(1-{\left(\xi_{j}\right)}^{q}\right)}^{w_{j}}}{\prod_{j=1}^{n}{\left(1+\left(\gamma -1\right){\left(\xi_{j}\right)}^{q}\right)}^{w_{j}}+\left(\gamma -1\right)\prod_{j=1}^{n}{\left(1-{\left(\xi_{j}\right)}^{q}\right)}^{w_{j}}}},\\ \frac{\sqrt[{q}]{\gamma }\prod_{j=1}^{n}{\left(\zeta_{j}\right)}^{w_{j}}}{\sqrt[{q}]{\prod_{j=1}^{n}{\left(1+\left(\gamma -1\right)\left(1-{\left(\zeta_{j}\right)}^{q}\right)\right)}^{w_{j}}+\left(\gamma -1\right)\prod_{j=1}^{n}{\left(\zeta_{j}\right)}^{qw_{j}}}} \end{array}\right) \end{array}$$

(13) $$\begin{array}{l} \mathrm{q-ROFHWG}\left(\Psi_{1},\Psi_{2},\ldots ,\Psi_{n}\right)=\overset{n}{\underset{j=1}{⊗}}{\left(\Psi_{j}\right)}^{\omega_{j}}\\ =\left(\begin{array}{l} \frac{\sqrt[{q}]{\gamma }\prod_{j=1}^{n}{\left(\xi_{j}\right)}^{w_{j}}}{\sqrt[{q}]{\prod_{j=1}^{n}{\left(1+\left(\gamma -1\right)\left(1-{\left(\xi_{j}\right)}^{q}\right)\right)}^{w_{j}}+\left(\gamma -1\right)\prod_{j=1}^{n}{\left(\xi_{j}\right)}^{qw_{j}}}},\\ \sqrt[{q}]{\frac{\prod_{j=1}^{n}{\left(1+\left(\gamma -1\right){\left(\zeta_{j}\right)}^{q}\right)}^{w_{j}}-\prod_{j=1}^{n}{\left(1-{\left(\zeta_{j}\right)}^{q}\right)}^{w_{j}}}{\prod_{j=1}^{n}{\left(1+\left(\gamma -1\right){\left(\zeta_{j}\right)}^{q}\right)}^{w_{j}}+\left(\gamma -1\right)\prod_{j=1}^{n}{\left(1-{\left(\zeta_{j}\right)}^{q}\right)}^{w_{j}}}} \end{array}\right) \end{array}$$

## 4. Determining the Attribute Weight Based on the q-ROFE

The fuzzy entropy is an important measure for depicting fuzziness and uncertain information, which has drawn numerous scholars’ attention in the past few years. Xu and Hu [43] defined the intuitionistic fuzzy entropy to determine an attribute’s weights and studied the entropy application for MADM. Chen et al. [44] proposed the interval-valued intuitionistic fuzzy entropy and applied it to actual firepower disposition issues. Ye [45] developed the interval-valued intuitionistic fuzzy cross-entropy for MADM. Xu and Xia [46] studied fuzzy entropy and cross-entropy under a hesitant fuzzy setting and applied it to MADM based on the TOPSIS method. Wei [47] presented picture fuzzy cross-entropy. Ye [48] proposed dual hesitant cross-entropy to manage actual MADM issues. Lu et al. [49] defined a TOPSIS method for PL-MAGDM with entropy weights. Gou et al. [50] defined some novel entropy and cross-entropy for hesitant fuzzy linguistic variables. Hu et al. [51] developed several similarity and entropy measures for hesitant fuzzy sets. To denote the entropy of the Pythagorean fuzzy set (PFS), Xue et al. [52] first developed the Pythagorean fuzzy entropy and the interval-valued Pythagorean fuzzy entropy based on the similarity measure and hesitance measure; then, a Pythagorean fuzzy entropy-based LINMAP method was proposed for a railway project investment problem. Yang and Hussain [53] also defined several Pythagorean fuzzy entropy for MADM. Thus, motivated by intuitionistic entropy and Pythagorean fuzzy entropy, in this study, the q-rung orthopair fuzzy entropy (q-ROFE) was defined as follows.

According to the Pythagorean fuzzy entropy shown in Xue et al. [52], we found that the entropy was mainly based on the similarity and hesitance measures, where the similarity measure between the q-rung orthopair fuzzy numbers $\Psi_{i}=\left(\xi_{i},\zeta_{i}\right)$ and its complement $\Psi_{i}^{C}=\left(\zeta_{i},\xi_{i}\right)$ can be denoted as:

(14) $$1-d\left(\Psi_{i},\Psi_{i}^{C}\right)=1-\left|\xi_{\Psi }^{q}\left(x_{i}\right)-\zeta_{\Psi }^{q}\left(x_{i}\right)\right|$$

In particular, when the membership and non-membership of the q-rung orthopair fuzzy number is equal, denoted as $\xi_{\Psi }^{}\left(x_{i}\right)=\zeta_{\Psi }^{}\left(x_{i}\right)$, this indicates the system is greatly disordered and we can obtain little useful information from the q-ROFN; thus, q−ROFE(Ψ)=1, if $\xi_{\Psi }^{}\left(x_{i}\right)=\zeta_{\Psi }^{}\left(x_{i}\right)$.

In addition, when the hesitancy of q-ROFN is equal to 1, denoted as $\pi_{\Psi }^{}\left(x_{i}\right)=1$, we can also barely derive valuable information from the q-ROFN; thus, q−ROFE(Ψ)=1, if $\pi_{\Psi }^{}\left(x_{i}\right)=1$.

Then, based on the relationship between q-ROFE and the similarity and hesitance measures, the computing equation can be defined.

**Definition** **8.** *Given a group of q-ROFNs as $\Psi_{i}=\left(\xi_{i},\zeta_{i}\right)\left(i=1,2,\ldots ,n\right)$, then the q-rung orthopair fuzzy entropy (q-ROFE) is defined as:*

(15) $$\begin{array}{ll} q-ROFE\left(\Psi_{i}\right) & =1-\left|\xi_{\Psi }^{q}\left(x_{i}\right)-\zeta_{\Psi }^{q}\left(x_{i}\right)\right|+\pi_{\Psi }^{q}\left(x_{i}\right)-\pi_{\Psi }^{q}\left(x_{i}\right)\left[1-\left|\xi_{\Psi }^{q}\left(x_{i}\right)-\zeta_{\Psi }^{q}\left(x_{i}\right)\right|\right]\\ & =1-\left(\xi_{\Psi }^{q}\left(x_{i}\right)+\zeta_{\Psi }^{q}\left(x_{i}\right)\right)\left|\xi_{\Psi }^{q}\left(x_{i}\right)-\zeta_{\Psi }^{q}\left(x_{i}\right)\right| \end{array}$$

*Thus,*

(16) $$q-ROFE\left(\Psi \right)=\frac{1}{n}\sum_{i=1}^{n}\left[1-\left(\xi_{\Psi }^{q}\left(x_{i}\right)+\zeta_{\Psi }^{q}\left(x_{i}\right)\right)\left|\xi_{\Psi }^{q}\left(x_{i}\right)-\zeta_{\Psi }^{q}\left(x_{i}\right)\right|\right]$$

Based on the basic axiom of fuzzy entropy, the q-rung orthopair fuzzy entropy (q-ROFE) will satisfy the following properties:

(1) 0≤q−ROFE(Ψ)≤1;
(2) q−ROFE(Ψ)=0, if Ψ is a crisp set;
(3) q−ROFE(Ψ)=1, if $\xi_{\Psi }^{q}\left(x_{i}\right)=\zeta_{\Psi }^{q}\left(x_{i}\right)$, ∀x∈X;
(4) $q-ROFE\left(\Psi_{1}\right)<q-ROFE\left(\Psi_{2}\right)$, if $\Psi_{1}$ is less fuzzy than $\Psi_{2}$, i.e., $\zeta_{\Psi_{1}}\left(x_{i}\right)\geq \zeta_{\Psi_{2}}\left(x_{i}\right)$ and $\xi_{\Psi_{1}}\left(x_{i}\right)\leq \xi_{\Psi_{2}}\left(x_{i}\right)$ for $\xi_{\Psi_{2}}\left(x_{i}\right)\leq \zeta_{\Psi_{2}}\left(x_{i}\right)$ for ∀x∈X, or $\zeta_{\Psi_{1}}\left(x_{i}\right)\leq \zeta_{\Psi_{2}}\left(x_{i}\right)$ and $\xi_{\Psi_{2}}\left(x_{i}\right)\geq \zeta_{\Psi_{2}}\left(x_{i}\right)$ for $\xi_{\Psi_{2}}\left(x_{i}\right)\geq \zeta_{\Psi_{2}}\left(x_{i}\right)$ for ∀x∈X;
(5) $q-ROFE\left(\Psi \right)=q-ROFE\left(\Psi^{C}\right)$.

**Proof.** 

(1) For $0\leq \xi_{\Psi }^{q}\left(x_{i}\right)+\zeta_{\Psi }^{q}\left(x_{i}\right)\leq 1$ and $0\leq \left|\xi_{\Psi }^{q}\left(x_{i}\right)-\zeta_{\Psi }^{q}\left(x_{i}\right)\right|\leq 1$, we can derive $0\leq 1-\left(\xi_{\Psi }^{q}\left(x_{i}\right)+\zeta_{\Psi }^{q}\left(x_{i}\right)\right)\left|\xi_{\Psi }^{q}\left(x_{i}\right)-\zeta_{\Psi }^{q}\left(x_{i}\right)\right|\leq 1$, thus 0≤q−ROFE(Ψ)≤1 is proved.
(2) If Ψ is a crisp set, which indicates Ψ=(1,0) or Ψ=(0,1), then q−ROFE(Ψ)=0; if q−ROFE(Ψ)=0, we can derive $\left(\xi_{\Psi }^{q}\left(x_{i}\right)+\zeta_{\Psi }^{q}\left(x_{i}\right)\right)\left|\xi_{\Psi }^{q}\left(x_{i}\right)-\zeta_{\Psi }^{q}\left(x_{i}\right)\right|=1$; for $0\leq \xi_{\Psi }^{q}\left(x_{i}\right)+\zeta_{\Psi }^{q}\left(x_{i}\right)\leq 1$ and $0\leq \left|\xi_{\Psi }^{q}\left(x_{i}\right)-\zeta_{\Psi }^{q}\left(x_{i}\right)\right|\leq 1$, then $\xi_{\Psi }^{q}\left(x_{i}\right)+\zeta_{\Psi }^{q}\left(x_{i}\right)=1$ and $\left|\xi_{\Psi }^{q}\left(x_{i}\right)-\zeta_{\Psi }^{q}\left(x_{i}\right)\right|=1$; for $0\leq \xi_{\Psi }\left(x_{i}\right)\leq 1$ and $0\leq \zeta_{\Psi }\left(x_{i}\right)\leq 1$, we can get $\xi_{\Psi }\left(x_{i}\right)=1,\zeta_{\Psi }\left(x_{i}\right)=0$ or $\xi_{\Psi }\left(x_{i}\right)=0,\zeta_{\Psi }\left(x_{i}\right)=1$, which means Ψ is a crisp set.
(3) If $\xi_{\Psi }^{q}\left(x_{i}\right)=\zeta_{\Psi }^{q}\left(x_{i}\right)$, then q−ROFE(Ψ)=1. If q−ROFE(Ψ)=1, we can obtain $\left(\xi_{\Psi }^{q}\left(x_{i}\right)+\zeta_{\Psi }^{q}\left(x_{i}\right)\right)\left|\xi_{\Psi }^{q}\left(x_{i}\right)-\zeta_{\Psi }^{q}\left(x_{i}\right)\right|=0$, which indicates $\left(\xi_{\Psi }^{q}\left(x_{i}\right)+\zeta_{\Psi }^{q}\left(x_{i}\right)\right)=0$ or $\left|\xi_{\Psi }^{q}\left(x_{i}\right)-\zeta_{\Psi }^{q}\left(x_{i}\right)\right|$, then we can obtain $\xi_{\Psi }^{q}\left(x_{i}\right)=\zeta_{\Psi }^{q}\left(x_{i}\right)$.
(4) If $\Psi_{1}$ is less fuzzy than $\Psi_{2}$, assuming that $\zeta_{\Psi_{1}}\left(x_{i}\right)\geq \zeta_{\Psi_{2}}\left(x_{i}\right)$ and $\xi_{\Psi_{1}}\left(x_{i}\right)\leq \xi_{\Psi_{2}}\left(x_{i}\right)$ for $\xi_{\Psi_{2}}\left(x_{i}\right)\leq \zeta_{\Psi_{2}}\left(x_{i}\right)$ for ∀x∈X, we can obtain:
  $$\begin{array}{l} q-ROFE\left(\Psi_{2}\right)-q-ROFE\left(\Psi_{1}\right)\\ =\frac{1}{n}\sum_{i=1}^{n}\left[1-\left(\xi_{\Psi_{2}}^{q}\left(x_{i}\right)+\zeta_{\Psi_{2}}^{q}\left(x_{i}\right)\right)\left|\xi_{\Psi_{2}}^{q}\left(x_{i}\right)-\zeta_{\Psi_{2}}^{q}\left(x_{i}\right)\right|\right]\\ -\frac{1}{n}\sum_{i=1}^{n}\left[1-\left(\xi_{\Psi_{1}}^{q}\left(x_{i}\right)+\zeta_{\Psi_{1}}^{q}\left(x_{i}\right)\right)\left|\xi_{\Psi_{1}}^{q}\left(x_{i}\right)-\zeta_{\Psi_{1}}^{q}\left(x_{i}\right)\right|\right]\\ =\frac{1}{n}\sum_{i=1}^{n}\left[\begin{array}{l} \left(\xi_{\Psi_{1}}^{q}\left(x_{i}\right)+\zeta_{\Psi_{1}}^{q}\left(x_{i}\right)\right)\left|\xi_{\Psi_{1}}^{q}\left(x_{i}\right)-\zeta_{\Psi_{1}}^{q}\left(x_{i}\right)\right|\\ -\left(\xi_{\Psi_{2}}^{q}\left(x_{i}\right)+\zeta_{\Psi_{2}}^{q}\left(x_{i}\right)\right)\left|\xi_{\Psi_{2}}^{q}\left(x_{i}\right)-\zeta_{\Psi_{2}}^{q}\left(x_{i}\right)\right| \end{array}\right]\\ =\frac{1}{n}\sum_{i=1}^{n}\left[\begin{array}{l} \left(\xi_{\Psi_{1}}^{q}\left(x_{i}\right)+\zeta_{\Psi_{1}}^{q}\left(x_{i}\right)\right)\left(\zeta_{\Psi_{1}}^{q}\left(x_{i}\right)-\xi_{\Psi_{1}}^{q}\left(x_{i}\right)\right)\\ -\left(\xi_{\Psi_{2}}^{q}\left(x_{i}\right)+\zeta_{\Psi_{2}}^{q}\left(x_{i}\right)\right)\left(\zeta_{\Psi_{2}}^{q}\left(x_{i}\right)-\xi_{\Psi_{2}}^{q}\left(x_{i}\right)\right) \end{array}\right]\\ =\frac{1}{n}\sum_{i=1}^{n}\left[\zeta_{\Psi_{1}}^{2q}\left(x_{i}\right)-\xi_{\Psi_{1}}^{2q}\left(x_{i}\right)-\zeta_{\Psi_{2}}^{2q}\left(x_{i}\right)+\xi_{\Psi_{2}}^{2q}\left(x_{i}\right)\right]\\ =\frac{1}{n}\sum_{i=1}^{n}\left[\begin{array}{l} \left(\zeta_{\Psi_{1}}^{q}\left(x_{i}\right)+\zeta_{\Psi_{2}}^{q}\left(x_{i}\right)\right)\left(\zeta_{\Psi_{1}}^{q}\left(x_{i}\right)-\zeta_{\Psi_{2}}^{q}\left(x_{i}\right)\right)\\ +\left(\xi_{\Psi_{2}}^{q}\left(x_{i}\right)+\xi_{\Psi_{1}}^{q}\left(x_{i}\right)\right)\left(\xi_{\Psi_{2}}^{q}\left(x_{i}\right)-\xi_{\Psi_{1}}^{q}\left(x_{i}\right)\right) \end{array}\right] \end{array}$$
  Since $0\leq \zeta_{\Psi_{1}}^{q}\left(x_{i}\right)-\zeta_{\Psi_{2}}^{q}\left(x_{i}\right)\leq 1$ and $0\leq \xi_{\Psi_{2}}^{q}\left(x_{i}\right)-\xi_{\Psi_{1}}^{q}\left(x_{i}\right)\leq 1$, then we can get $q-ROFE\left(\Psi_{2}\right)-q-ROFE\left(\Psi_{1}\right)\geq 0$, which indicates $q-ROFE\left(\Psi_{1}\right)<q-ROFE\left(\Psi_{2}\right)$; similarly, if $\Psi_{2}$ is less fuzzy than $\Psi_{1}$, then $q-ROFE\left(\Psi_{2}\right)<q-ROFE\left(\Psi_{1}\right)$.
(5) For a q-rung orthopair fuzzy complement set $\Psi^{C}$, the entropy can be depicted as:
  $$\begin{array}{l} q-ROFE\left(\Psi^{C}\right)=\frac{1}{n}\sum_{i=1}^{n}\left[1-\left(\zeta_{\Psi }^{q}\left(x_{i}\right)+\xi_{\Psi }^{q}\left(x_{i}\right)\right)\left|\zeta_{\Psi }^{q}\left(x_{i}\right)-\xi_{\Psi }^{q}\left(x_{i}\right)\right|\right]\\ =\frac{1}{n}\sum_{i=1}^{n}\left[1-\left(\xi_{\Psi }^{q}\left(x_{i}\right)+\zeta_{\Psi }^{q}\left(x_{i}\right)\right)\left|\xi_{\Psi }^{q}\left(x_{i}\right)-\zeta_{\Psi }^{q}\left(x_{i}\right)\right|\right]=q-ROFE\left(\Psi \right) \end{array}$$
  Thus, the property $q-ROFE\left(\Psi \right)=q-ROFE\left(\Psi^{C}\right)$ is maintained. Therefore, all the properties are proved. □

On account of the q-ROFE, we can determine the attribute’s weights. Suppose there are m alternatives $\phi_{i}\left(i=1,2,\ldots ,m\right)$ and each alternative is denoted by n attributes $\varepsilon_{j}\left(j=1,2,\ldots ,m\right)$. Construct the q-rung orthopair fuzzy evaluation matrix $R_{ij}={\left(r_{ij}\right)}_{m\times n}$; if the attribute’s weights is unknown, then we can determine the attribute’s weights using the following steps.

Step 1. Compute the q-ROFE of each element in matrix $R_{ij}={\left(r_{ij}\right)}_{m\times n}$, and construct a q-ROFE matrix as follows:

(17) $$q-ROFE_{ij}={\left[\begin{array}{cccc} q-ROFE_{11} & q-ROFE_{12} & \cdots & q-ROFE_{1n}\\ q-ROFE_{21} & q-ROFE_{22} & \cdots & q-ROFE_{2n}\\ \vdots & \vdots & \ddots & \vdots \\ q-ROFE_{m1} & q-ROFE_{m2} & \cdots & q-ROFE_{mn} \end{array}\right]}_{m\times n}$$

Step 2. Normalize the q-ROFE matrix to derive the normalized matrix $q-ROFNE_{ij}$ using the following equation:

(18) $$q-ROFNE_{ij}=\frac{q-ROFE_{ij}}{\max\left(q-ROFE_{i1},q-ROFE_{i2},\ldots ,q-ROFE_{in}\right)}$$

Step 3. Determine the attribute’s weights $w_{j}\left(j=1,2,\ldots ,n\right)$ based on the normalized matrix $q-ROFNE_{ij}$ using the following equation:

(19) $$w_{j}=\frac{1-\sum_{i=1}^{m}q-ROFNE_{ij}}{n-\sum_{i=1}^{m}\sum_{j=1}^{n}q-ROFNE_{ij}}\left(j=1,2,\ldots ,n\right)$$

## 5. The Entropy-Based GLDS Method for MAGDM with q-ROFN Information

The gained and lost dominance score (GLDS) method, which was first proposed by Wu and Liao [32], is used to choose the most desirable alternative(s) by calculating the dominance flow between any two alternatives with respect to the attributes. The higher the gained dominance score is and the lower the lost dominance score is, the best the alternative will be. Wu and Liao [32] studied the consensus-based GLDS method under a probabilistic linguistic environment for selecting the best green enterprises. Then, Fu et al. [33] extended the GLDS method to hesitant fuzzy linguistic term sets and developed the hesitant fuzzy linguistic GLDS method for an underground mining method selection problem. In this study, based on the q-ROFS, we proposed to combine the GLDS method with q-ROFN, where the basic MAGDM steps are as follows.

Suppose there are m given alternatives $\phi_{i}\left(i=1,2,\ldots ,m\right)$ and each alternative is denoted by n given attributes $\varepsilon_{j}\left(j=1,2,\ldots ,m\right)$. Let $w_{j}\left(j=1,2,\ldots ,m\right)$ be the attribute weighting vector that satisfies $0\leq w_{j}\leq 1,\sum_{j=1}^{n}w_{j}=1$. Construct the q-rung orthopair fuzzy decision-making evaluation matrix $R_{ij}={\left(r_{ij}\right)}_{m\times n}$. Then, the q-rung orthopair fuzzy GLDS method can be developed as follows.

Step 1. Compute the dominance flow $DF_{j}\left(\phi_{i},\phi_{k}\right)$ of alternative $\phi_{i}$ over $\phi_{k}$ with respect to the attributes $\varepsilon_{j}$ using the following equation:

(20) $$DF_{j}\left(\phi_{i},\phi_{k}\right)=\left\{ \begin{array}{l} \max\left\{\tau \left(r_{ij}\right)-\tau \left(r_{kj}\right),0\right\},\;\mathrm{for}\;\mathrm{benefit}\;\mathrm{attribute}\;\varepsilon_{j}\\ \max\left\{\tau \left(r_{kj}\right)-\tau \left(r_{ij}\right),0\right\},\;\mathrm{for}\;\cos t\;\mathrm{attribute}\;\varepsilon_{j} \end{array}\right.$$

where τ(r) indicates the function of converting the q-rung orthopair fuzzy variables to the exact numbers. To eliminate the biased information derived using different attribute values, normalize the dominance flows $DF_{j}\left(\phi_{i},\phi_{k}\right)$ using the following equation:

(21) $$NDF_{j}\left(\phi_{i},\phi_{k}\right)=\frac{DF_{j}\left(\phi_{i},\phi_{k}\right)}{\sqrt{\sum_{k=1}^{m}\sum_{i=1}^{m}{\left(DF_{j}\left(\phi_{i},\phi_{k}\right)\right)}^{2}}}$$

Step 2. According to the normalized dominance flows $NDF_{j}\left(\phi_{i},\phi_{k}\right)$, we can compute the overall gained dominance scores $OGDS\left(\phi_{i}\right)$ of alternative $\phi_{i}$ over $\phi_{k}$ with respect to the attributes $\varepsilon_{j}$ using the following equation and derive the subordinate order set $R_{1}$:

(22) $$OGDS\left(\phi_{i}\right)=\sum_{j=1}^{n}\left(w_{j}\sum_{k=1}^{m}NDF_{j}\left(\phi_{i},\phi_{k}\right)\right)$$

Step 3. According to the normalized dominance flows $NDF_{j}\left(\phi_{i},\phi_{k}\right)$, we can compute the overall lost dominance scores $OLDS\left(\phi_{i}\right)$ of alternative $\phi_{i}$ over $\phi_{k}$ with respect to the attributes $\varepsilon_{j}$ using the following equation and derive the subordinate order set $R_{2}$:

(23) $$OLDS\left(\phi_{i}\right)=\underset{j}{\max}\left(w_{j}\underset{k}{\max}\left(NDF_{j}\left(\phi_{i},\phi_{k}\right)\right)\right)$$

Step 4. Normalize the subordinate order sets $OGDS\left(\phi_{i}\right)$ and $OLDS\left(\phi_{i}\right)$ to obtain the normalized subordinate order sets $NOGDS\left(\phi_{i}\right)$ and $NOLDS\left(\phi_{i}\right)$ using the following equations:

(24) $$NOGDS\left(\phi_{i}\right)=\frac{OGDS\left(\phi_{i}\right)}{\sqrt{\sum_{i=1}^{m}{\left(OGDS\left(\phi_{i}\right)\right)}^{2}}}$$

(25) $$NOLDS\left(\phi_{i}\right)=\frac{OLDS\left(\phi_{i}\right)}{\sqrt{\sum_{i=1}^{m}{\left(OLDS\left(\phi_{i}\right)\right)}^{2}}}$$

Step 5. On account of the two subordinate order sets ($R_{1}$ and $R_{2}$) and the two normalized score sets ($NOGDS\left(\phi_{i}\right)$ and $NOLDS\left(\phi_{i}\right)$), we can compute the final results $CS_{i}$ to rank all the alternatives using the following equation:

(26) $$CS_{i}=NOGDS\left(\phi_{i}\right)\cdot \frac{m-R_{1}\left(\phi_{i}\right)+1}{m\left(m+1\right)/2}-NOLDS\left(\phi_{i}\right)\cdot \frac{R_{2}\left(\phi_{i}\right)}{m\left(m+1\right)/2}$$

Based on the above computing steps, we can see that the GLDS method is robust and effective. First, the final calculating equation can not only consider the gained and lost scores, but also take the subordinate order set into account. Then, the GLDS method can consider both the “group utility” (see Equation (22)) and the “individual regret” (see Equation (23)); in addition, the GLDS method can be easily extended to other decision-making environments, including quantitative and qualitative environments. Finally, in the decision-making process, we utilize the normalization method two times to derive more accurate results and accelerate the decision-making speed.

## 6. The MAGDM Steps Based on the q-rung Orthopair Fuzzy Entropy-Based GLDS Method

Suppose there are m given alternatives $\phi_{i}\left(i=1,2,\ldots ,m\right)$ and each alternative is denoted by n attributes $\varepsilon_{j}\left(j=1,2,\ldots ,m\right)$. Let $w_{j}\left(j=1,2,\ldots ,m\right)$ be the attribute weighting vector, which satisfies $0\leq w_{j}\leq 1,\sum_{j=1}^{n}w_{j}=1$. Assume that there are λ experts $d_{t}\left(t=1,2,\ldots ,\lambda \right)$ with an expert’s weighting vector of $\omega_{t}\left(t=1,2,\ldots ,\lambda \right)$, which satisfies $0\leq \omega_{t}\leq 1,\sum_{t=1}^{\lambda }\omega_{t}=1$. Construct the q-rung orthopair fuzzy evaluation matrix $R_{ij}^{t}={\left(r_{ij}^{t}\right)}_{m\times n}$. Then, the q-rung orthopair fuzzy entropy-based GLDS method can be developed as follows.

Step 1. Collect the assessment information expressed using q-ROFNs that are given by experts $d_{t}\left(t=1,2,\ldots ,\lambda \right)$ and construct the decision-making evaluation matrix $R_{ij}^{t}={\left(r_{ij}^{t}\right)}_{m\times n}$.

Step 2. Based on the expert’s weights and decision-making evaluation matrix $R_{ij}^{t}={\left(r_{ij}^{t}\right)}_{m\times n}$, aggregate the assessment information to derive the comprehensive evaluation matrix $R_{ij}^{}={\left(r_{ij}^{}\right)}_{m\times n}$ using Equation (12) or (13).

Step 3. According to the comprehensive evaluation matrix $R_{ij}^{}={\left(r_{ij}^{}\right)}_{m\times n}$, compute the q-rung orthopair fuzzy entropy (q-ROFE) using Equation (16) and then determine the attribute weights using Equations (17)–(19).

Step 4. Compute the dominance flows $DF_{j}\left(\phi_{i},\phi_{k}\right)$ of alternative $\phi_{i}$ over $\phi_{k}$ with respect to the attributes $\varepsilon_{j}$ using Equations (4) and (20), and obtain the normalized dominance flow $NDF_{j}\left(\phi_{i},\phi_{k}\right)$ using Equation (21).

Step 5. Determine the overall gained dominance scores $OGDS\left(\phi_{i}\right)$ and the overall lost dominance score $OLDS\left(\phi_{i}\right)$ using Equations (22) and (23), and derive the subordinate order set $R_{1}$ and $R_{2}$. Then, normalize Equations (22) and (23) to obtain Equations (24) and (25).

Step 6. On account of the two-subordinate-order sets ($R_{1}$ and $R_{2}$) and the two-normalized-score sets ($NOGDS\left(\phi_{i}\right)$ and $NOLDS\left(\phi_{i}\right)$), we can compute the final results $CS_{i}$ to rank all the alternatives using Equation (26).

Step 7. End.

## 7. Numerical Example and Comparative Analysis

### 7.1. Numerical Example 

The economic development of China has currently entered a "new normal," which means the China has left the past era of extensive and high-growth and turned to a stage of intensive structural and high-quality development. As the times require, a PPP model emerged as an important link for pushing the supply-side structural reform forward and improving the efficiency of the public service supply. Within only 4 years, PPP has entered the fast lane of rapid development and become the mainstream model of infrastructure investment in China since the formal promotion of the National Committee in 2014. By the end of 2018, up to 12,554 PPP projects were uploaded to the database of the Ministry of Finance, totaling ¥17.54 trillion in investment. With the development of PPP, many problems have gradually emerged. On the one hand, because of the immature PPP financing market and few financing channels, investors face difficulties obtaining financial support, leading to the low project implementation rate. On the other hand, the huge market stock is difficult to revitalize due to the poor exit channels, which causes increasing concerns regarding social capital investment. To eliminate these negative aspects that affect and restrict the PPP development, in 2017, the National Development and Reform Commission and the Ministry of Finance issued papers in succession to encourage PPP securitization. As a way of structured financing, securitization can provide flexible and diversified standardized products, connect the main body of PPP project with the main body of multi-investment effectively, and promote the optimal allocation of project resources. PPP securitization of China is still in the initial exploration stage such that successful cases are relatively few, the existing studies are mainly qualitative analyses, and the research dimension is relatively singular. Analyses have failed to produce an in-depth discussion of the PPP securitization operation mechanism and are unable to pay sufficient attention to the influencing factors of the PPP securitization success in particular. Thus, how to choose the PPP project is an interesting MAGDM issue [54,55,56,57,58,59,60,61]. In this section, a numerical example for the social capital selection of a PPP project is given with q-ROFNs to demonstrate the method proposed in this paper. There were five possible PPP projects $\phi_{i}\left(i=1,2,3,4,5\right)$ to select. Three experts selected four attributes to evaluate the five PPP projects: (1) $\varepsilon_{1}$ is the financial capacity, (2) $\varepsilon_{2}$ is the technical ability, (3) $\varepsilon_{3}$ is the management ability, and (4) $\varepsilon_{4}$ is the reputation level. The five possible PPP projects $\phi_{i}\left(i=1,2,3,4,5\right)$ were evaluated by the decision-maker in terms of the above four attributes using the q-rung orthopair fuzzy information (the expert’s weighting vector was considered to be $\omega ={\left(0.4,0.2,0.4\right)}^{T}$).

Step 1. The assessment information expressed by q-ROFNs, which were given by experts $d_{t}\left(t=1,2,\ldots ,\lambda \right)$, were collected and used to construct the decision-making evaluation matrix $R_{ij}^{t}={\left(r_{ij}^{t}\right)}_{m\times n}$:

$$R^{1}=\left[\begin{array}{cccc} \left(0.7,0.6\right) & \left(0.5,0.4\right) & \left(0.6,0.3\right) & \left(0.4,0.7\right)\\ \left(0.8,0.5\right) & \left(0.4,0.6\right) & \left(0.5,0.4\right) & \left(0.3,0.6\right)\\ \left(0.9,0.7\right) & \left(0.6,0.8\right) & \left(0.7,0.5\right) & \left(0.8,0.4\right)\\ \left(0.5,0.3\right) & \left(0.4,0.2\right) & \left(0.6,0.3\right) & \left(0.2,0.5\right)\\ \left(0.4,0.6\right) & \left(0.3,0.5\right) & \left(0.7,0.3\right) & \left(0.6,0.8\right) \end{array}\right],$$

$$R^{2}=\left[\begin{array}{cccc} \left(0.5,0.4\right) & \left(0.6,0.5\right) & \left(0.7,0.2\right) & \left(0.5,0.6\right)\\ \left(0.7,0.6\right) & \left(0.5,0.8\right) & \left(0.6,0.3\right) & \left(0.4,0.5\right)\\ \left(0.8,0.4\right) & \left(0.6,0.2\right) & \left(0.5,0.7\right) & \left(0.6,0.3\right)\\ \left(0.6,0.5\right) & \left(0.3,0.5\right) & \left(0.7,0.5\right) & \left(0.6,0.4\right)\\ \left(0.5,0.8\right) & \left(0.7,0.4\right) & \left(0.6,0.2\right) & \left(0.7,0.3\right) \end{array}\right],$$

$$R^{3}=\left[\begin{array}{cccc} \left(0.6,0.7\right) & \left(0.3,0.8\right) & \left(0.7,0.4\right) & \left(0.5,0.6\right)\\ \left(0.5,0.8\right) & \left(0.2,0.5\right) & \left(0.6,0.3\right) & \left(0.7,0.4\right)\\ \left(0.6,0.5\right) & \left(0.7,0.5\right) & \left(0.4,0.6\right) & \left(0.6,0.5\right)\\ \left(0.7,0.4\right) & \left(0.9,0.4\right) & \left(0.7,0.3\right) & \left(0.2,0.8\right)\\ \left(0.8,0.7\right) & \left(0.3,0.6\right) & \left(0.4,0.7\right) & \left(0.7,0.2\right) \end{array}\right].$$

Step 2. Based on the expert’s weights and decision-making evaluation matrix $R_{ij}^{t}={\left(r_{ij}^{t}\right)}_{m\times n}$, the assessment information was aggregated to obtain the comprehensive evaluation matrix $R_{ij}^{}={\left(r_{ij}^{}\right)}_{m\times n}$ using the q-ROFHWA operator:

$$R=\left[\begin{array}{cccc} \left(0.6287,0.5933\right) & \left(0.4683,0.5656\right) & \left(0.6638,0.3109\right) & \left(0.4647,0.6394\right)\\ \left(0.6892,0.6354\right) & \left(0.3759,0.5964\right) & \left(0.5639,0.3369\right) & \left(0.5405,0.4941\right)\\ \left(0.7974,0.5522\right) & \left(0.6439,0.5202\right) & \left(0.5712,0.5775\right) & \left(0.6970,0.4139\right)\\ \left(0.6128,0.3736\right) & \left(0.7075,0.3186\right) & \left(0.6638,0.3330\right) & \left(0.3623,0.5895\right)\\ \left(0.6368,0.6794\right) & \left(0.4437,0.5160\right) & \left(0.5893,0.3973\right) & \left(0.6638,0.3945\right) \end{array}\right].$$

Step 3. According to the comprehensive evaluation matrix $R_{ij}^{}={\left(r_{ij}^{}\right)}_{m\times n}$, the q-rung orthopair fuzzy entropy (q-ROFE) was computed using Equation (16):

$$q-ROFE_{ij}=\left[\begin{array}{cccc} 0.9819 & 0.9778 & 0.9154 & 0.9418\\ 0.9586 & 0.9578 & 0.9693 & 0.9896\\ 0.7713 & 0.9485 & 0.9976 & 0.8904\\ 0.9498 & 0.8756 & 0.9158 & 0.9603\\ 0.9684 & 0.9888 & 0.9620 & 0.9183 \end{array}\right].$$

Then, the attribute weights were determined using Equations (17)–(19): $w_{1}=0.2448,w_{2}=0.2525,w_{3}=0.2533,w_{4}=0.2494$.

Step 4. The dominance flows $DF_{j}\left(\phi_{i},\phi_{k}\right)$ of alternative $\phi_{i}$ over $\phi_{k}$ with respect to the attributes $\varepsilon_{j}$ was computed using Equations (4) and (20). If all the attributes are beneficial, the dominance flow can be derived as follows:

$$DF_{1}\left(\phi_{i},\phi_{k}\right)=\left[\begin{array}{l} 0.0000\;0.0000\;0.0000\;0.0000\;0.0475\\ 0.0156\;0.0000\;0.0000\;0.0000\;0.0631\\ 0.1495\;0.1339\;0.0000\;0.0803\;0.1970\\ 0.0692\;0.0536\;0.0000\;0.0000\;0.1167\\ 0.0000\;0.0000\;0.0000\;0.0000\;0.0000 \end{array}\right],$$

$$DF_{2}\left(\phi_{i},\phi_{k}\right)=\left[\begin{array}{l} 0.0000\;0.0404\;0.0000\;0.0000\;0.0000\\ 0.0000\;0.0000\;0.0000\;0.0000\;0.0000\\ 0.1022\;0.1426\;0.0000\;0.0000\;0.0881\\ 0.2000\;0.2404\;0.0978\;0.0000\;0.1859\\ 0.0141\;0.0545\;0.0000\;0.0000\;0.0000 \end{array}\right],$$

$$DF_{3}\left(\phi_{i},\phi_{k}\right)=\left[\begin{array}{l} 0.0000\;0.0606\;0.1343\;0.0034\;0.0602\\ 0.0000\;0.0000\;0.0737\;0.0000\;0.0000\\ 0.0000\;0.0000\;0.0000\;0.0000\;0.0000\\ 0.0000\;0.0572\;0.1309\;0.0000\;0.0568\\ 0.0000\;0.0004\;0.0741\;0.0000\;0.0000 \end{array}\right],$$

$$DF_{4}\left(\phi_{i},\phi_{k}\right)=\left[\begin{array}{l} 0.0000\;0.0000\;0.0000\;0.0000\;0.0000\\ 0.0992\;0.0000\;0.0000\;0.0973\;0.0000\\ 0.2143\;0.1152\;0.0000\;0.2125\;0.0183\\ 0.0019\;0.0000\;0.0000\;0.0000\;0.0000\\ 0.1960\;0.0969\;0.0000\;0.1942\;0.0000 \end{array}\right].$$

Step 5. The normalized dominance flows $NDF_{j}\left(\phi_{i},\phi_{k}\right)$ was derived using Equation (21) and the overall gained dominance scores $OGDS\left(\phi_{i}\right)$ and the overall lost dominance scores $OLDS\left(\phi_{i}\right)$ were determined using Equations (22) and (23). Then, the subordinate order sets $R_{1}$ and $R_{2}$ were derived as follows.

The overall gained dominance scores $OGDS\left(\phi_{i}\right)$ were:

$$\begin{array}{l} OGDS\left(\phi_{1}\right)=0.3259,OGDS\left(\phi_{2}\right)=0.2405,OGDS\left(\phi_{3}\right)=0.9085,\\ OGDS\left(\phi_{4}\right)=0.8533,OGDS\left(\phi_{5}\right)=0.3828. \end{array}$$

The overall lost dominance scores $OLDS\left(\phi_{i}\right)$ were:

$$\begin{array}{l} OLDS\left(\phi_{1}\right)=0.1390,OLDS\left(\phi_{2}\right)=0.0762,OLDS\left(\phi_{3}\right)=0.1433,\\ OLDS\left(\phi_{4}\right)=0.1410,OLDS\left(\phi_{5}\right)=0.1068. \end{array}$$

Thus, the subordinate order sets $R_{1}$ and $R_{2}$ were: $R_{1}=\left(0.3259,0.2405,0.9085,0.8533,0.3828\right)$ and $R_{2}=\left(0.1390,0.0762,0.1433,0.1410,0.1068\right)$. Then, the $OGDS\left(\phi_{i}\right)$ and $OLDS\left(\phi_{i}\right)$ were normalized using Equations (24) and (25) to obtain the following.

The normalized overall gained dominance scores $NOGDS\left(\phi_{i}\right)$ were:

$$\begin{array}{l} NOGDS\left(\phi_{1}\right)=0.2387,NOGDS\left(\phi_{2}\right)=0.1762,NOGDS\left(\phi_{3}\right)=0.6654,\\ NOGDS\left(\phi_{4}\right)=0.6250,NOGDS\left(\phi_{5}\right)=0.2804. \end{array}$$

The normalized overall lost dominance scores $NOLDS\left(\phi_{i}\right)$ were:

$$\begin{array}{l} NOLDS\left(\phi_{1}\right)=0.1390,NOLDS\left(\phi_{2}\right)=0.0762,NOLDS\left(\phi_{3}\right)=0.1433,\\ NOLDS\left(\phi_{4}\right)=0.1410,NOLDS\left(\phi_{5}\right)=0.1068. \end{array}$$

Step 6. On account of the two subordinate order sets ($R_{1}$ and $R_{2}$) and two normalized score sets ($NOGDS\left(\phi_{i}\right)$ and $NOLDS\left(\phi_{i}\right)$), we computed the final results $CS_{i}$: $CS_{1}=0.0856,$$CS_{2}=0.0663,CS_{3}=0.2209,CS_{4}=0.2097,CS_{5}=0.1022.$ Then, we obtained the ordering of all PPP projects as $\phi_{3}>\phi_{4}>\phi_{5}>\phi_{1}>\phi_{2}$, where the most desirable PPP project was $\phi_{3}$.

### 7.2. Comparative Analysis 

To further verify the effective and scientific nature of our proposed approach, in this part, we shall compare the q-rung orthopair fuzzy entropy-based GLDS method with other existing methods, such as the q-ROFWA and q-ROFWG operators presented by Liu and Wang [21] and the q-rung orthopair fuzzy cosine similarity measures given in Wang et al. [62]. The comparative analysis process is listed below.

#### 7.2.1. Comparison with the q-ROFWA and q-ROFWG Operators

Based on the comprehensive evaluation matrix $R_{ij}^{}={\left(r_{ij}^{}\right)}_{m\times n}$ and the attribute weights $w_{j}$ derived using the q-ROFE, we aggregated the comprehensive assessment information using the q-ROFWA and q-ROFWG operators. The fused results $r_{i}$ are listed as follows.

For the q-ROFWA operator, the fused results $r_{i}$ were:

$$\begin{array}{l} r_{1}=\left(0.5754,0.5071\right),r_{2}=\left(0.5691,0.5001\right),r_{3}=\left(0.6940,0.5120\right),\\ r_{4}=\left(0.6213,0.3906\right),r_{5}=\left(0.5979,0.4831\right). \end{array}$$

Then, based on the score functions, we obtained: $SC\left(r_{1}\right)=0.5300,SC\left(r_{2}\right)=0.5296,$ $SC\left(r_{3}\right)=0.6000,SC\left(r_{4}\right)=0.5901,SC\left(r_{5}\right)=0.5505.$ The ordering of all PPP projects was $\phi_{3}>\phi_{4}>\phi_{5}>\phi_{1}>\phi_{2}$, where the most desirable PPP project was $\phi_{3}$.

For the q-ROFWG operator, the fused results $r_{i}$ were:

$$\begin{array}{l} r_{1}=\left(0.5487,0.5582\right),r_{2}=\left(0.5290,0.5432\right),r_{3}=\left(0.6713,0.5247\right),\\ r_{4}=\left(0.5688,0.4386\right),r_{5}=\left(0.5759,0.5304\right). \end{array}$$

Then, based on the score functions, we obtained: $SC\left(r_{1}\right)=0.4957,SC\left(r_{2}\right)=0.4939,$$SC\left(r_{3}\right)=0.5791,SC\left(r_{4}\right)=0.5498,SC\left(r_{5}\right)=0.5209.$ The ordering of all PPP projects was $\phi_{3}>\phi_{4}>\phi_{5}>\phi_{1}>\phi_{2}$, where the most desirable alternative is $\phi_{3}$.

From the above comparative analysis results, by utilizing the q-ROFWA and q-ROFWG operators, we obtained the same rank of all alternatives as that derived using the q-rung-orthopair-fuzzy-entropy-based GLDS method, which indicates that our developed approach is effective at managing actual MADM problems. However, the q-ROFWA and q-ROFWG operators can only derive the score results to rank all alternatives and fail at reflecting the dominance flow of the alternatives over the attributes. In our developed q-rung orthopair fuzzy entropy-based GLDS method, the dominance flow can be reflected using step 4. Moreover, Liu and Wang’s methods do not take the unknown weights into account; in their paper, the attribute weights were assumed to be known but this is unrealistic in a real MAGDM environment. The q-rung-orthopair-fuzzy-entropy-based GLDS method can overcome this limitation since the attribute weights can be derived using fuzzy entropy, which is reasonable and scientific.

#### 7.2.2. Comparison with the q-Rung Orthopair Fuzzy Cosine Similarity Measures

Based on the comprehensive evaluation matrix $R_{ij}^{}={\left(r_{ij}^{}\right)}_{m\times n}$, we obtained the ideal solution alternative $\phi^{+}$ by utilizing the methods shown in Wang et al. [62] as follows:

$$\phi^{+}=\left\{\left(0.7974,0.3736\right),\left(0.7075,0.3186\right),\left(0.6638,0.3109\right),\left(0.6970,0.3945\right)\right\}.$$

Then, based on attribute weights $w_{j}$ derived using the q-ROFE and the q-rung orthopair fuzzy cosine similarity measures, we derived:

$$\begin{array}{l} q-ROFWCS\left(\phi_{1},\phi^{+}\right)\\ =\left[\begin{array}{l} 0.2448\times \cos\left[\frac{\pi }{4}\left(\left|0.6287^{3}-0.7974^{3}\right|+\left|0.5933^{3}-0.3736^{3}\right|\right)\right]\\ +0.2525\times \cos\left[\frac{\pi }{4}\left(\left|0.4683^{3}-0.7075^{3}\right|+\left|0.5656^{3}-0.3186^{3}\right|\right)\right]\\ +0.2533\times \cos\left[\frac{\pi }{4}\left(\left|0.6638^{3}-0.6638^{3}\right|+\left|0.3109^{3}-0.3109^{3}\right|\right)\right]\\ +0.2494\times \cos\left[\frac{\pi }{4}\left(\left|0.4647^{3}-0.6970^{3}\right|+\left|0.6394^{3}-0.3945^{3}\right|\right)\right] \end{array}\right]\\ =0.9601 \end{array}.$$

Similarly, we obtained:

$$\begin{array}{l} q-ROFWCS\left(\phi_{2},\phi^{+}\right)=0.9656,q-ROFWCS\left(\phi_{3},\phi^{+}\right)=0.9904,\\ q-ROFWCS\left(\phi_{4},\phi^{+}\right)=0.9798,q-ROFWCS\left(\phi_{5},\phi^{+}\right)=0.9686. \end{array}$$

Thus, the ordering of all PPP projects was $\phi_{3}>\phi_{4}>\phi_{5}>\phi_{2}>\phi_{1}$, where the most deriarable PPP project was $\phi_{3}$. 

From the above comparative analysis results, according to the the q-rung orthopair fuzzy cosine similarity measures, the ordering was slightly different than that found using the q-rung-orthopair-fuzzy-entropy-based GLDS method, which indicates that our developed approach is effective at managing actual MAGDM problems. However, the q-rung orthopair fuzzy cosine similarity measures are also limited in their reflection of the dominance flow of the alternatives over the attributes and consideration of the unknown weights, which is in contrast with our method over the q-rung orthopair fuzzy cosine similarity measures.

#### 7.2.3. Comparison with Other Existing Methods

In addition, numerous scholars have studied the q-ROFSs, such as the BM [27,28] operator, Heronian mean (HM) [29,30,63] operator, Hamy mean (HM) [64] operator and MSM [22] operator. The contribution of different authors regarding q-ROFNs are listed in Table 1. All of these methods can handle MADM problems with q-ROFSs and select the best alternatives using score results, but when we utilized these mentioned methods, the DM’s and attribute’s weights needed to be completely known, and at the same time, the process of decision-making was only up to a decision-maker. However, due to the complex environment and the subjectivity of the decision-maker, the decision-making process is always uncertain and the weights information is partly known or completely unknown. When we face such issues, the q-ROFE developed in this paper can be more useful for managing MADM applications. Furthermore, to obtain more accurate and effective decision-making results, the evaluation information is given using three DMs in this paper rather than only one DM in other existing literature.

## 8. Conclusions

In this paper, a q-rung orthopair fuzzy entropy-based GLDS method was developed to manage MAGDM issues. Considering the inter-relationship between the q-ROFNs, we developed the q-ROFHWA and q-ROFHWG operators to fuse the assessment information. Then, because the attribute weights are usually partly known or completely unknown in complicated application environments, we proposed a q-rung orthopair fuzzy entropy to determine the attribute weights. Next, to depict the dominance flow of alternatives over attributes, the GLDS method was put forward to solve q-rung orthopair fuzzy MAGDM issues. Therefore, the q-rung-orthopair-fuzzy-entropy-based GLDS model was built based on a q-ROFHWA operator, q-ROFE, and a traditional GLDS method. In the end, we took a concrete case about the social capital selection of PPP projects to demonstrate our defined model and verify its accuracy. The main advantage of our method was the consideration of the dominance flow and the unknown weights, which indicates that our method is valid for managing actual decision-making issues. However, in our method, the dominance flow is simply derived using the difference value between score results, which will lead to information loss; thus, in the future, we shall continue to study the MAGDM issues with our developed method and find a more suitable way to describe the dominance flow in other decision-making domains [65,66,67,68,69,70].

## Figures and Tables

**Table 1:** The contribution of different authors regarding q-ROFNs.

Authors	Production	Consider the Interrelationship	Consider the Parameter Vector	Consider the Dominance Flow	Consider the Unknown Weights
Liu and Wang [21]	q-ROFWA operator	No	No	No	No
Liu and Wang [21]	q-ROFWG operator	No	No	No	No
Wei et al. [22]	q-ROFMSM operators	Yes	Yes	No	No
Bai et al. [23]	q-ROF-partitioned-MSM operators	Yes	Yes	No	No
Liu et al. [24]	q-ROF-power-MSM operators	Yes	Yes	No	No
Liu et al. [25]	q-ROFEBM operators	Yes	Yes	No	No
Liu and Liu [26]	q-ROFBM operators	Yes	Yes	No	No
Liu and Liu [27]	Lq-ROF-power-BM operators	Yes	Yes	No	No
Yang and Pang [28]	q-ROF-partitioned-BM operators	Yes	Yes	No	No
Wei et al. [29]	q-R2TLOFHM operators	Yes	Yes	No	No
Liu et al. [30]	q-ROFHM operators	Yes	Yes	No	No
Xu et al. [63]	q-RDHOFHM operators	Yes	Yes	No	No
Proposed model	Entropy-based GLDS method	Yes	Yes	Yes	Yes

q-ROFWA operator: q-rung orthopair fuzzy weighted averaging operator; q-ROFWG operator: q-rung orthopair fuzzy weighted geometric operator; q-ROFMSM operators: q-rung orthopair fuzzy Maclaurin symmetric mean operator; q-ROF-partitioned-MSM operators: q-rung orthopair fuzzy partitioned Maclaurin symmetric mean operator; q-ROF-power-MSM operators: q-rung orthopair fuzzy power Maclaurin symmetric mean operator; q-ROFEBM operators: q-rung orthopair fuzzy extended Bonferroni mean; q-ROFBM operators: q-rung orthopair fuzzy Bonferroni mean operators; Lq-ROF-power-BM operators: linguistic q-rung orthopair fuzzy power Bonferroni mean operators; q-ROF-partitioned-BM operators: q-rung orthopair fuzzy partitioned Bonferroni mean operators; q-R2TLOFHM operators: q-rung 2-tuple linguistic orthopair fuzzy Heronian mean operators; q-ROFHM operators: q-rung orthopair fuzzy Heronian mean operators; q-RDHOFHM operators: q-rung dual hesitant Fuzzy orthopair fuzzy Heronian mean operators; Entropy-based GLDS method: Entropy-based gained and lost dominance score method.
